# Targeting Lactate Dehydrogenase-B as a Strategy to Fight Cancer: Identification of Potential Inhibitors by In Silico Analysis and In Vitro Screening

**DOI:** 10.3390/pharmaceutics15102411

**Published:** 2023-10-01

**Authors:** Manos Vlasiou, Vicky Nicolaidou, Christos Papaneophytou

**Affiliations:** 1 Department of Veterinary Medicine, University of Nicosia School of Veterinary Medicine, 2414 Nicosia, Cyprus; vlasiou.m@unic.ac.cy; 2 Department of Life Sciences, School of Life and Health Sciences, University of Nicosia, 2417 Nicosia, Cyprus; nicolaidou.v@unic.ac.cy

**Keywords:** lactate dehydrogenase-B, cancer, inhibitors, colorimetric assay, molecular dynamics

## Abstract

Lactate dehydrogenase (LDH) is an enzyme that catalyzes the reversible conversion of lactate to pyruvate while reducing NAD^+^ to NADH (or oxidizing NADH to NAD^+^). Due to its central role in the Warburg effect, LDH-A isoform has been considered a promising target for treating several types of cancer. However, research on inhibitors targeting LDH-B isoform is still limited, despite the enzyme’s implication in the development of specific cancer types such as breast and lung cancer. This study aimed to identify small-molecule compounds that specifically inhibit LDH-B. Our in silico analysis identified eight commercially available compounds that may affect LDH-B activity. The best five candidates, namely tucatinib, capmatinib, moxidectin, rifampicin, and acetyldigoxin, were evaluated further in vitro. Our results revealed that two compounds, viz., tucatinib and capmatinib, currently used for treating breast and lung cancer, respectively, could also act as inhibitors of LDH-B. Both compounds inhibited LDH-B activity through an uncompetitive mechanism, as observed in in vitro experiments. Molecular dynamics studies further support these findings. Together, our results suggest that two known drugs currently being used to treat specific cancer types may have a dual effect and target more than one enzyme that facilitates the development of these types of cancers. Furthermore, the results of this study could be used as a new starting point for identifying more potent and specific LDH-B inhibitors.

## 1. Introduction

Cancer is characterized by abnormal cell growth supported by alterations in metabolic pathways and the tumor microenvironment [1]. Cancer cells rely heavily on glucose and exhibit a phenotype of upregulated glucose transport and metabolism known as the Warburg effect [2,3]. While most cancers generate ATP via anaerobic glycolysis, some types, including breast and lung cancers, exhibit metabolic heterogeneity [4] and rely on autophagy to promote cell survival and proliferation [5]. These metabolic and molecular alterations provide potential targets for cancer treatment.

LDH is a promising target for cancer therapy [6]. LDH is an enzyme complex consisting of four subunits, comprising two different isoforms: LDH-A (M isoform, encoded by the *Ldha* gene) and LDH-B (H isoform, encoded by the *Ldhb* gene). LDH-A plays a crucial role in the glycolytic pathway due to its higher affinity for pyruvate, and it catalyzes the conversion of pyruvate to lactate by oxidizing NADH to NAD^+^ [7]. On the contrary, LDH-B shows a greater affinity for lactate and converts lactate to pyruvate by reducing NAD^+^ to NADH [8,9]. Elevated pyruvate concentrations can strongly inhibit LDH-B activity, a phenomenon known as the “substrate-inhibition effect” [10]. LDH isoenzymes differ in their proportion of LDH-A and LDH-B subunits and tissue distribution [11]. LDH tetramers form a total of five isoenzymes: LDH-1 (4H), LDH-2 (3H1M), LDH-3 (2H2M), LDH-4 (1H3M), and LDH-5 (4M) [11]. There are also two additional LDH genes, namely *Ldhc* and *Ldhd*, which are primarily expressed in the testes [8] and are not within the scope of this work.

The role of LDH in cancer metabolism is highlighted in several studies (reviewed in [1,12]). Other studies compared the kinetic parameters of LDH between cancer and normal cells. For example, Talaiezadeh et al. [13] demonstrated that LDH isolated from cancer cells had higher K_m_ for both lactate and NAD^+^ when compared with normal LDH. On the contrary, De Bari et al. [14] and Pizzuto et al. [15] have shown that the K_m_ values for lactate and NAD^+^ did not significantly differ between cancer cell lines (Prostate PC3 and HEP G2) and normal cells. The above data indicate that cancer cells require a particular environment to grow and proliferate.

In contrast, the microenvironment of tumor tissue imposes various limitations that force cancer cells to alter their metabolism to persist and continue to proliferate. Furthermore, Talaiezadeh et al. [13] suggested that high lactate concentrations have a slight inhibitory effect on LDH in cancer cells as it has a higher affinity for lactate, and therefore, the enzyme can tolerate higher lactate concentrations. The preferential catalysis of lactate to pyruvate by LDH in cancer cells may be an adaptive response to the increased lactate levels commonly present in the tumor microenvironment (TME) [12]. Furthermore, studies have reported significantly higher lactate levels in metastatic tumors compared to non-metastatic tumors in patients [16]. Together, the above data suggest that LDH plays a significant role in the glucose metabolism of cancer cells and can impact both tumorigenesis and metastasis.

Although the involvement of LDH-A in cancer initiation and progression has been extensively investigated, the contribution of LDH-B to cancer metabolism remains insufficiently explored [12]. Upregulation of LDH-B in tumors has been correlated with disease progression and poor prognosis [17,18,19,20]. However, the link between cancer and LDH-B expression is intricate, as promoter methylation leads to the silencing of LDH-B in multiple cancers [21]. However, increased LDH-B expression has been observed in various adenocarcinomas, lung cancer, and breast cancer, particularly in highly aggressive and metastatic cancer types [12]. LDH-B and other glycolysis-related enzymes are overexpressed in basal-like triple-negative breast cancer (TNBC) compared with different breast cancer subtypes. This upregulation coincides with inferior clinical outcomes [22]. Thus, LDH-B has been considered a potential target for treating these types of cancer. The primary function of LDH-B in cancer metabolism is to convert lactate to pyruvate, allowing cancer cells to generate energy and biosynthetic precursors more efficiently, which is essential for their growth and survival [12]. As previously mentioned, LDH-B converts lactate and NAD^+^ into pyruvate, NADH, and H^+^ ions. These H^+^ ions stimulate lysosomal acidification and autophagy in cancer; however, the exact mechanism of this function remains inconclusive [23]. Furthermore, it has been suggested that lactate is a key metabolic player in cancer [24].

Therefore, LDH-B expression and activity measurement can be used as a biomarker for cancer diagnosis and prognosis, and its role in cancer metabolism is highlighted in several recent works [25,26,27,28]. Interestingly, clinical evaluation of LDH-B could be a predictive marker of response for patients with breast cancer receiving neoadjuvant chemotherapy [29]. It has been suggested that targeting LDH-B may be a potential therapeutic strategy for cancer treatment, as it could inhibit cancer cell growth and survival [1]. Despite the prominent role of LDH-B in breast and lung cancer metabolism, there are only a few known inhibitors of this enzyme. For example, oxamate, a structural analog of pyruvate, is a competitive inhibitor of both LDH-A and LDH-B, and it has been the subject of numerous studies that have shown interesting anticancer effects on various cancer cell lines, including those obtained from HCC, breast cancer, CRC, lymphoma, medulloblastoma, and ovarian cancer [30,31,32,33,34,35]. Moreover, recently, Shibita et al. [36] reported the first specific inhibitor of human LDH-B, namely AXKO-0046.

Several inhibitors are currently being evaluated in vitro and in vivo for their effectiveness against LDH-A, with or without activity against LDH-B (reviewed in [12]). However, research aimed at identifying potent and specific inhibitors of LDH-B remains limited. This study aimed to identify small-molecule compounds explicitly targeting LDH-B and investigate the mechanism underlying their inhibitory effect. The results of this work may serve as a starting point for developing more specific and potent LDH-B inhibitors.

## 2. Materials and Methods

### 2.1. Materials

Unless otherwise stated, all chemicals were purchased from Sigma-Aldrich (Saint Louis, MO, USA). Recombinant human LDH-B was purchased from R&D Systems (Minneapolis, MN, USA) (Catalog #: 9205-HB). The LDH-B candidate inhibitors (Table 1) were purchased from Cayman Chemical (Ann Arbor, MI, USA) (Catalog #: tucatinib: 31411, capmatinib:20056; moxidectin: 17165; rifampicin: 14423; acetyldigoxin: 22266). AXKO-0046 was obtained from MedChemExpress (Monmouth Junction, NJ, USA) (Catalog #: HY-147216A). The purity of all compounds was higher than 90%. It should be noted that the compounds bictegravir, calicheamicin, and capreomycin were not evaluated in vitro.

Nitro blue tetrazolium (NBT) and phenazine methosulfate (PMS) stock solutions (30 mM) were prepared in 70% *v*/*v* dimethylformamide (DMF) and distilled water, respectively, and protected from light.

### 2.2. Methods

#### 2.2.1. Virtual Screening

To find the candidate inhibitors, first, we screened “the zinc database”, a free database for commercially available compounds for virtual screening (https://zinc.docking.org (accessed on 1 February 2023 )). After having a potential use of 1,825,113,472 substances, we narrowed our choices using the filters (FOR SALE/BIOACTIVE), and 2528 possible inhibitors were used for virtual screening. Virtual screening was performed using the software Autodock Vina v.1.1.2 (The Scripps Research Institute, La Jolla, CA, USA) [37,38]. To proceed with the molecular docking studies, we used the human B lactate dehydrogenase structure from the protein data bank (1T2F: PDB) [39]. Docking was carried out on PyRx using the AutoDock Vina option and ran at an ‘exhaustiveness’ of 8. The grid box was centered at X = 12.1477, Y = −3.5864, and Z = 18.4151, with a grid dimension of 45.0279 Å × 68.7439 Å × 56.9456 Å, thereby enclosing both the active site residues and the binding site. This procedure narrowed our choices to eight substances with the best binding affinity scores (−10.0) [40]. We performed ligand-based screening through the CHEMBL_act library (SwissSimilarity) to add more options to the work. Using the threshold of 0.9 probability of likeness, 32 more candidate molecules were added to the 40 candidate inhibitor molecules. After conducting several ligand–receptor docking runs, the Vina software analyzed the outcomes, determined the binding affinities of the ligands, and grouped the resulting poses based on their conformations. The top ligands were then verified by re-docking them into the same predetermined regions of the receptor using AutoDock Vina, based on their binding affinities. The resulting re-docked complex was compared to the reference co-crystallized complex through superimposition, and the root mean square deviation (RMSD) was calculated. The best eight candidates (Table 1) were further evaluated, and three compounds were excluded from further analysis as described in the results in Section 3.1, “In silico screening of potential LDH-B inhibitors”. The effect of the remaining five compounds on LDH-B activity was assessed in vitro, as described in the following paragraphs.

#### 2.2.2. Molecular Dynamics

The best candidates were subjected to further examination of their inhibitory effects through molecular dynamics studies. In detail, molecular dynamics simulations of the best candidates were performed as a second validation method using the AMBER force fields [41]. The complexes were placed in a rectangular parallel-piped water box, and an explicit solvent model for water was used while the complexes were solvated with a 10 Å water cap. Chlorine ions were added as counterions to neutralize the system. Before the MD simulations, one step of minimization was carried out. The MD trajectories were run using the minimized structures as the starting conformations. The time step of the simulations was 2.0 fs, with a cutoff of 10 Å for the non-bonded interactions. Constant-volume periodic boundary MD was carried out for 300 ps. The temperature was raised from 0 to 300 K. All ligands that showed an average RMSD greater than 2 Å (Supplementary Table S1) concerning the reference disposition were discarded using the docking result as a reference pose [42].

#### 2.2.3. Molecular Docking

The structure-based pharmacophore modeling was performed by molecular docking using the iGEMDOCK software v 2.1 (Institute of Bioinformatics, National Chiao Tung University, Hsinchu, Taiwan) [43]. Docking preparation was carried out as previously described [44]. Ligand molecules are used for pharmacophore modeling. We used CHIMERA software v.1.17 (Resource for Biocomputing, Visualization, and Informatics, University of California, San Francisco, USA) to prepare the protein structure by assigning the hydrogen atoms, charges, and energy minimization [45]. The energy minimization was performed using the 500 steepest descent steps with a 0.02 Å step size and an update interval of 10. This study concluded with the ten best candidates in total.

#### 2.2.4. LDH-B Activity Assay

The activity of LDH-B was determined using an endpoint colorimetric assay in a 96-well format, as we previously described in [46]. In brief, LDH-B converts lactate to pyruvate by reducing NAD^+^ to NADH. The released NADH reacts with NBT, which, in the presence of PMS, forms a blue-purple formazan that exhibits maximum absorption at ~570 nm. The assay was carried out in reaction buffer consisting of 50 mM CHES buffer, pH 9.6, supplemented with 150 mM NaCl, 300 μΜ ΝΒΤ, 30 μΜ PMS, and 0.13% gelatin to avoid the precipitation of formazan [47]. Reaction mixtures were prepared in the wells of a 96-well microtiter plate. Initially, 90 μL of reaction buffer was added to each well. This was followed by the addition of 25 μL of substrate mix (10 mM NAD^+^ and 250 mM sodium lactate in 50 mM CHES buffer, pH 9.6, containing 150 mM NaCl). Then, 5 μL of the inhibitor solution at various concentrations was added to achieve the desired concentration, as indicated in Section 3, “Results” In the control samples, 5 μL of buffer was added instead of the inhibitor solution. The plate was incubated (in the dark) at 25 °C for 30 s, and the absorbance ($A_{initial}^{570nm}$) was measured at 570 nm in a microplate reader (PerkinElmer, Waltham, MA, USA). The reaction was initiated by adding two (2) μL of a 7.5 μΜ LDH-B solution. The plate was incubated for 5 min at 25 °C in the dark under continuous shaking, and subsequently, the absorbance at 570 nm was measured for a second time ($A_{final}^{570nm}$). The amount of NADH that was produced was determined using the ΔA_570 nm_ = ($A_{final}^{570nm}$ ^-^$A_{initial}^{570nm})$ from a standard curve (0–15 nmole of NADH) [46]. All absorbance values were corrected using a blank sample without LDH-B. One unit of enzyme activity was defined as the amount of enzyme required to liberate 1 μmol of NADH/min at 25 °C. LDH-B activity (mU/mL) was determined using Equation (1):

(1) $$LDH\;activity(\frac{mU}{mL})=\frac{\left[NADH](nmol\right)}{\left(T_{final-}T_{initial}\right)(min)\times V(mL)}=\frac{nmol}{\min\times mL}$$

where: [NADH] is the amount (in nmol) of NADH that is released between T_final_ and T_initial,_ and V is the sample volume (LDH-B in mL) added in each well of the 96-well plate.

The IC_50_ values (i.e., the concentration of inhibitor required to increase the measured response to 50% of its maximal value) of tucatinib and capmatinib were calculated with GraphPad Prism v.8.0.2 (GraphPad Software Inc., San Diego, CA, USA) using the software’s built-in equations, based on the raw data obtained from the enzymatic assays. The IC_50_ values were obtained by fitting the dose–response curve using non-linear regression analysis.

To verify our observations, we next used a kinetic assay in cuvette form (with a final reaction volume of 3 mL) to determine LDH-B activity. This assay employs the same principle as the end-point assay described above. Briefly, the assay mixture was composed of 2 mL reaction buffer (comprising 50 mM CHES buffer, pH 9.6, supplemented with 150 mM NaCl, 300 μM NBT, 30 μM PMS, and 0.13% gelatin), 0.5 mL of 6 mM NAD^+^, and 0.5 mL of 150 mM sodium lactate. The reaction was initiated by adding 4 μL of LDH-B solution (to reach a final concentration of 7.5 nM). After mixing the samples, they were immediately loaded into a Jasco V-530 UV-Visible Spectrophotometer (Jasco, Easton, MD, USA). Absorbance was recorded every 20 s over 420 s, and changes in absorbance (ΔA570 nm) were determined. The amount of NADH produced was ascertained using a standard curve (0–15 nmol of NADH). All absorbance values were corrected using a blank sample without LDH-B. LDH activity was then determined using Equation (1) as described above.

All experiments were conducted in triplicate, with data presented as mean values ± standard deviation (S.D.).

#### 2.2.5. Zymogram Analysis

To further assess the effect of candidate inhibitors on LDH-B activity and investigate whether these compounds disrupt the LDH-B tetramer, we employed an NBT/PMS-based zymogram analysis, as previously described in [48], with some modifications.

As mentioned before, NBT, in the presence of PMS, reacts with NADH produced by dehydrogenases, resulting in the formation of an insoluble blue-purple formazan. This NBT-PMS reaction can be used to visualize LDH-B in polyacrylamide gels. LDH-B (5 μg) was incubated with various concentrations of the potential inhibitors (discussed further in the results) for 30 min and loaded on a 10% native polyacrylamide gel. We used LDH-B (5 μg) as a control, which was not treated with any of the inhibitors. The gels were run at 100 V under non-reducing conditions and subsequently washed with 50 mM CHES buffer, pH 9.6, containing 150 mM NaCl. The LDH-B bands were visualized by incubating the gel for 10 min at 25 °C in the reaction buffer containing NAD^+^ and sodium lactate to a final concentration of 1 mM and 25 mM, respectively. LDH-B (5 μg) was pretreated for 30 min with different concentrations of the two potential inhibitors and subsequently loaded on native gels and run under the same condition to examine whether the two inhibitors disrupted the LDH-B tetramer.

#### 2.2.6. Kinetic Studies

To identify the mechanism of LDH-B inhibition by tucatinib and capmatinib, which exhibited the highest inhibitory effect on LDH-B, we performed enzymatic assays in the presence of increasing concentrations of the substrate (lactate) or the cofactor (NAD^+^). The compounds’ inhibition mechanism was evaluated further using the classic Michaelis-Menten approach (kinetics studies). To this end, eight concentrations of NAD^+^ (120, 240, 360, 480, 700, 900, 1000, and 1200 μM) were incubated in the presence of 25 mM sodium lactate for NAD^+^ titration, while eight concentrations of sodium lactate (2.5, 5, 10, 15, 20, 25, 37.5, and 50 mM) were incubated in the presence of 1 mM NAD^+^ for sodium lactate concentration. Subsequently, a solution of either of the two compounds (200, 300, 400, 500, 600, 800, and 1000 μΜ) with 2.5 nΜ LDH-B was added and incubated for 6.5 min at room temperature. The relationship between the initial reaction velocity and substrate concentration for each inhibitor concentration was fitted to a nonlinear Michaelis–Menten equation to calculate the K_m_ and V_max_ values using GraphPad Prism v.8.0.2 (GraphPad Software Inc., San Diego, CA, USA). Lineweaver–Burk plots were created by plotting the 1/[Velocity] vs. 1/[Substrate] data values and the line corresponding to the Michaelis–Menten nonlinear fit.

Furthermore, the LDH inhibition data were analyzed using GraphPad Prism v.8.0.2 and the software’s built-in enzyme kinetics analyses. These datasets were plotted as substrate concentration versus enzyme activity, fitted with second-order polynomial regression, and analyzed using the mixed-model inhibition fit. This approach is preferred over Lineweaver–Burk plot analysis as it avoids the approximation errors associated with the transformation of experimental data [49]. The mixed-model fit is described by Equations (2)–(4) and contains competitive, uncompetitive, and noncompetitive inhibition terms:

(2) $$V_{max}^{app}=V_{max}/(1+\frac{I}{a\cdot K_{i}})$$

(3) $$K_{m}^{app}=K_{m}\cdot (1+\frac{I}{K_{i}})/(1+\frac{I}{a\cdot K_{i}})$$

(4) $$Y=V_{max}^{app}\cdot X/(K_{m}^{app}+X)$$

In the above equations, $V_{max}^{app}$ and $K_{m}^{app}$ are the maximum enzyme activity and Michaelis–Menten constant, respectively, in the presence of an inhibitor; V_max_ and K_m_ represent the maximum enzyme velocity, and Michaelis–Menten constant, respectively, in the absence of an inhibitor, K_m_, and K_i_ is the inhibition constant. The mechanism of action is determined by the alpha (α) value. In this calculation model, X denotes the substrate concentration, Y represents enzyme activity, and I indicates inhibitor concentration. The model also incorporates the parameter α, which can indicate the inhibition mechanism. The α value serves as an index of the extent to which the inhibitor binding alters the enzyme’s affinity for the substrate, i.e., the value of the α parameter determines the mechanism of inhibition. An α equal to 1 indicates a noncompetitive inhibitor; thus, the inhibitor has a similar affinity for both the free enzyme and the enzyme–substrate complex. A very high value of α indicates a competitive inhibitor, i.e., the inhibitor competes with the substrate for binding to the active site. When α < 1, the inhibitor preferentially binds to the enzyme–substrate complex (a minimal α value but greater than 0 indicates an uncompetitive inhibitor, i.e., the inhibitor binds with greater affinity to the enzyme–substrate complex).

#### 2.2.7. Statistical Analysis

Unless otherwise stated, experiments were carried out in triplicate, and the data are presented as mean values ± standard deviation (SD). Statistical analysis was performed using one-way ANOVA, followed by Dunnett’s test for multiple comparisons (Supplementary Figures S1 and S2). Statistical significance was set at *p* < 0.05. The analysis was conducted using GraphPad Prism v.8.0.2 (GraphPad Software Inc., San Diego, CA, USA).

## 3. Results

### 3.1. In Silico Screening of Potential LDH-B Inhibitors

**Figure 1:**
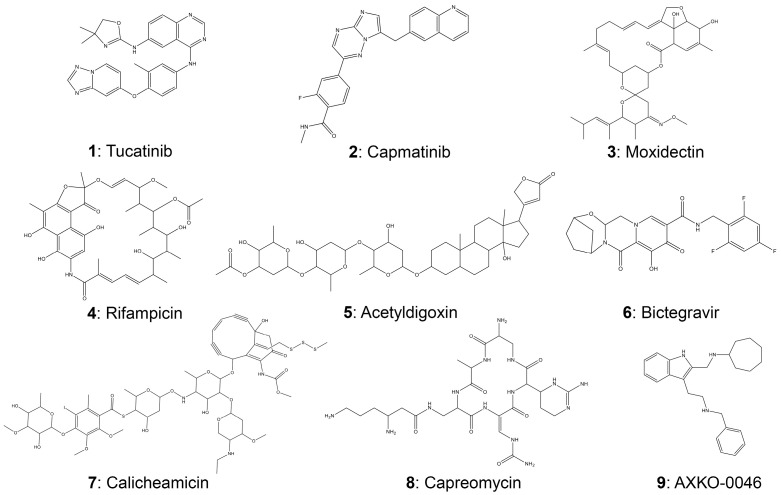
The chemical structures of the potential LDH-B inhibitors with the highest binding affinities after the virtual screening of 2528 compounds. AXKO-0046 (compound **9**) was used as a positive control.

We employed virtual screening using commercially available compounds to identify potential inhibitors that selectively target LDH-B. Through computer-aided drug discovery and virtual screening techniques, we identified eight compounds that may exhibit an inhibitory effect on LDH-B activity. The structures and names of these potential inhibitors are illustrated in Figure 1, and their selection process is described in detail in the “Methods” section. For comparison, we also included AXKO-0046 (N-((3-(2-(benzylamino)ethyl)-1H-indol-2-yl)methyl)cycloheptanamine) in this study (Figure 1; compound **9**). This indole derivative is currently the only well-characterized selective LDH-B inhibitor [36], which inhibits LDH-B via an uncompetitive mechanism with an EC_50_ value of 42 nM.

Table 1 summarizes the properties of the eight candidate inhibitors and AXKO-0046, including their cLogP, molecular mass, and virtual binding affinities.

We then searched PubChem (https://pubchem.ncbi.nlm.nih.gov/ (accessed on 6 February 2023)) for information about these compounds and published bioassay data. Compounds **1** (tucatinib) and **2** (capmatinib) are orally bioavailable drugs for treating specific cancer types. In detail, tucatinib is an inhibitor of the human epidermal growth factor receptor tyrosine kinase ErbB-2 (also called HER2) used in combination with other medications (i.e., trastuzumab and capecitabine) to treat unresectable or metastatic HER-2-positive breast cancer. Capmatinib is an inhibitor of the proto-oncogene c-Met (or hepatocyte growth factor receptor/HGFR) commonly used to treat non-small metastatic-cell lung cancer. On the other hand, moxidectin (compound **3**) is a macrocyclic lactone derived from *Streptomyces cyanogriseus* that exhibits antiparasitic activity. Rifampicin (compound **4**) is an ansamycin antibiotic used to treat various bacterial infections. Acetyldigoxin (compound **5**) is a natural compound found in the plant *Digitalis lanata* and used to treat congestive heart failure. Bictegravir (compound **6**) is a second-generation human immunodeficiency virus (HIV) integrase inhibitor and was excluded from further evaluation due to its low binding affinity. Unfortunately, no information is available in the PubChem database for compound **7** (calicheamicin). Despite this, according to Pubchem, calicheamicin has been linked to the onset of genetic diseases, which has led to its exclusion from further evaluation. Finally, capreomycin (compound **8**) is an injectable, broad-spectrum antibiotic used in combination with other antituberculosis drugs as a second-line treatment for drug-resistant tuberculosis. Furthermore, compound **8** was deemed not worth further study due to its high molecular mass (Lipinski rules violation). The inhibitory potential of the remaining five compounds was investigated further using in vitro assays, as discussed in the following paragraphs. Importantly, we wanted to further evaluate the potential of compounds **1** and **2** as dual inhibitors of enzymes that have been implicated in the development of specific cancer types, such as breast and lung cancer.

### 3.2. In Vitro Evaluation of Candidate LDH-B Inhibitors

We subsequently evaluated the inhibitory effect of the five best compounds using the in vitro colorimetry assay in a 96-well format we recently developed [46]. Our initial goal was to examine whether these compounds, at concentrations ranging from 100 to 2000 μM, affect the formation of the blue-purple formazan. Our findings indicated that none of the five compounds and AXKO-0046 disrupted the assay at the tested concentrations (Supplementary Figure S1). However, slight precipitation of all compounds was observed in wells with concentrations higher than 1200 μΜ. Therefore, subsequently, we evaluated the effect of the compounds on LDH-B activity at concentrations up to 1000 μΜ.

**Figure 2:**
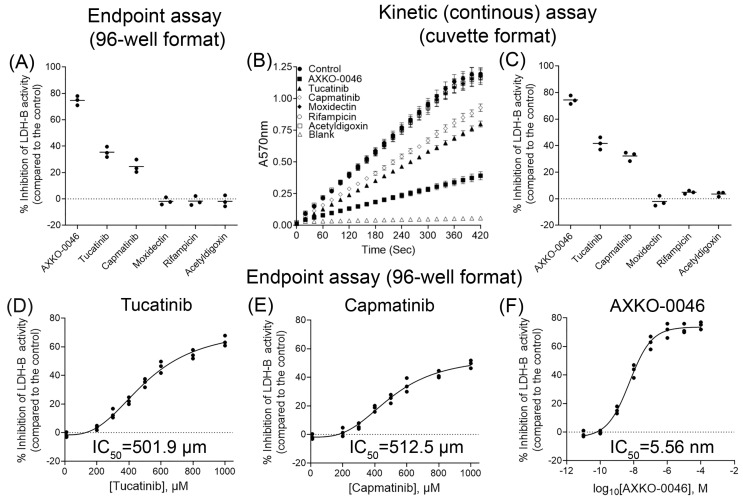
Screening of candidate inhibitors of LDH-B. (**A**) Effect of the five best candidates from virtual screening and positive control (AXKO-0046) on LDH-B activity. The inhibitory effect of the compounds was assessed in a 96-well format end-point colorimetric assay, as described in the text. (**B**,**C**)The effect of the five potential inhibitors and AXKO-0046 on LDH-B activity was also subsequently evaluated using a kinetic (continuous) colorimetric assay in a cuvette format, as described in the text. The changes in the absorbance at 570 nm (due to the formation of the formazan derivative) were monitored in the absence and presence of the inhibitors for 420 s (**B**). The continuous assay also confirmed that tucatinib and capmatinib inhibit LDH-B activity (**C**). Dose-dependent inhibitory effect of tucatinib (**D**), capmatinib (**E**), and AXKO-0046 (**F**) on LDH-B activity. In all (**A**,**C**–**F**), the % inhibitory effect of the candidate inhibitors was assessed by comparing LDH-B activity in the presence of each compound with that of a control (without any of the inhibitors). The results are presented as mean values ± S.D (n = 3).

We initially tested all compounds at a final concentration of 500 μΜ, and as shown in Figure 2A, only tucatinib and capmatinib inhibited LDH-B activity by approximately 35% and 24%, respectively. It should be noted that both compounds have not been reported as pan assay interference compounds (PAINS) [50]. Interestingly, acetyldigoxin, which exhibited the highest score in virtual screening, did not affect LDH-B activity. As expected, AXKO-0046 at the tested concentration inhibited LDH-B activity by approximately 75% (Figure 2A).

To verify the results obtained with the colorimetric assay in a 96-well format (end-point assay), we subsequently assessed the effects of the five best candidates and AXKO-0046 (positive control) on LDH-B activity using a kinetic assay in a cuvette-format (3 mL final volume), as described in Section 2.2.4, ‘LDH-B Activity Assay’. As shown in Figure 2B, in the absence of any of the tested inhibitors, there was an increase in absorbance at 570 nm, which was due to the formation of the formazan derivative. Notably, the assay remained linear for over 5 min, affirming the method’s robustness. In contrast, the inclusion of AXKO-0046, tucatinib, or capmatinib in the assay mixture resulted in a reduced rate of increase in absorbance at 570 nm (Figure 2B). Converting the changes in absorbance at 570 nm into enzymatic activity showed that AXKO-0046 inhibited LDH-B activity by more than 75%, while tucatinib and capmatinib inhibited the enzyme by approximately 43% and 31%, respectively. In alignment with the results from the end-point assay, the remaining three compounds (moxidectin, rifampicin, and acetyldigoxin) had a minor impact on LDH-B activity (Figure 2B,C). The slight observed discrepancies in the percentage inhibition of LDH-B activity (by AXKO-0046, tucatinib, and capmatinib) between the 96-well format assay (end-point assay) and the cuvette-format assay (kinetic assay) could be partly attributed to the differences in reaction volumes (~100 μL in the end-point assay vs. 3 mL in the kinetic assay).

Thus, we thoroughly examined the biological properties of the two best candidates, while the remaining three (moxidectin, rifamycin, and acetyldigoxin) were not subjected to further evaluation. We subsequently examined the effect of various concentrations, ranging from 100 to 1000 μΜ of tucatinib and capmatinib, on LDH-B activity using the end-point assay in a 96-well format. As illustrated in Figure 2D,E, both tucatinib and capmatinib showed concentration-dependent inhibition of LDH-B activity with IC_50_ values equal to 501.9 μΜ and 512.5 μΜ, respectively. To further assess the suitability of our assay in determining compounds that selectively inhibit LDH-B, we examined the effect of AXKO-0046 in the concentration range (10^−9^–10^−4^ M) previously examined by Shibata et al. [36]. As shown in Figure 2F and in agreement with the findings of Shibata et al. [36], AXKO-0046 inhibits LDH-B in a concentration-dependent manner with an IC_50_ value in the nanomolar range (IC_50_ = 5.56 nm). Together, the above results highlight the suitability of our colorimetric end-point assay for identifying compounds that selectively inhibit LDH-B. While tucatinib and capmatinib partially inhibited LDH-B activity at a relatively high concentration (at the μΜ levels), compared to AXKO-0046, which inhibits LDH-B at the nM range (discussed further in Section 4, “Discussion”), this inhibition was not attributable to compound concentration effects such as aggregation, instability, or assay interference.

**Figure 3:**
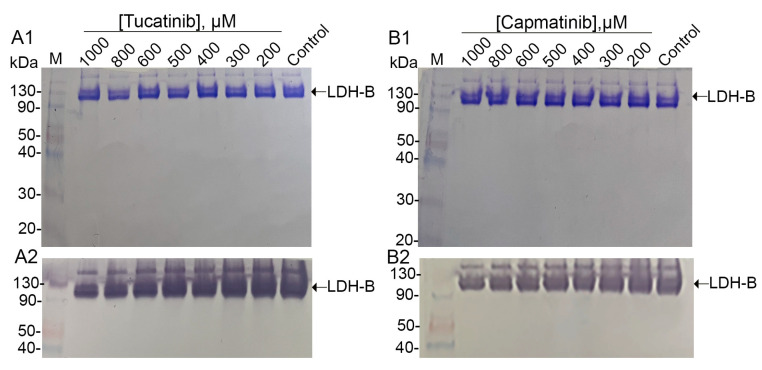
Effect of tucatinib and capmatinib on tetramer formation and activity of LDH-B. Equal amounts of LDH-B (5 ng) were incubated with increasing concentrations of either tucatinib (**A1**,**A2**) or capmatinib (**B1**,**B2**) for 30 min, followed by loading onto 10% native (non-reducing) polyacrylamide gels. After electrophoresis, gels were stained with Coomassie Brilliant Blue to assess the effect of both compounds on LDH-B tetramer formation (top panels). As shown, neither compound affected the integrity of the tetramer. A second set of gels underwent zymogram analysis, as described in the text, to assess the effect of the potential inhibitors on enzyme activity (bottom panels). LDH-B activity decreased with increasing concentrations of both compounds.

Since LDH-B forms a homotetrameric complex (LDH1) for lactate-to-pyruvate conversion, we investigated the impact of tucatinib and capmatinib on tetramer formation. Native PAGE analysis of LDH-B pre-incubated with both compounds revealed no effect on tetramer formation for either tucatinib or capmatinib (Figure 3A1,B1, respectively). However, zymogram analysis demonstrated decreased LDH-B activity with increasing concentrations of both compounds (Figure 3A2,B2, respectively).

### 3.3. Kinetic Studies

**Figure 4:**
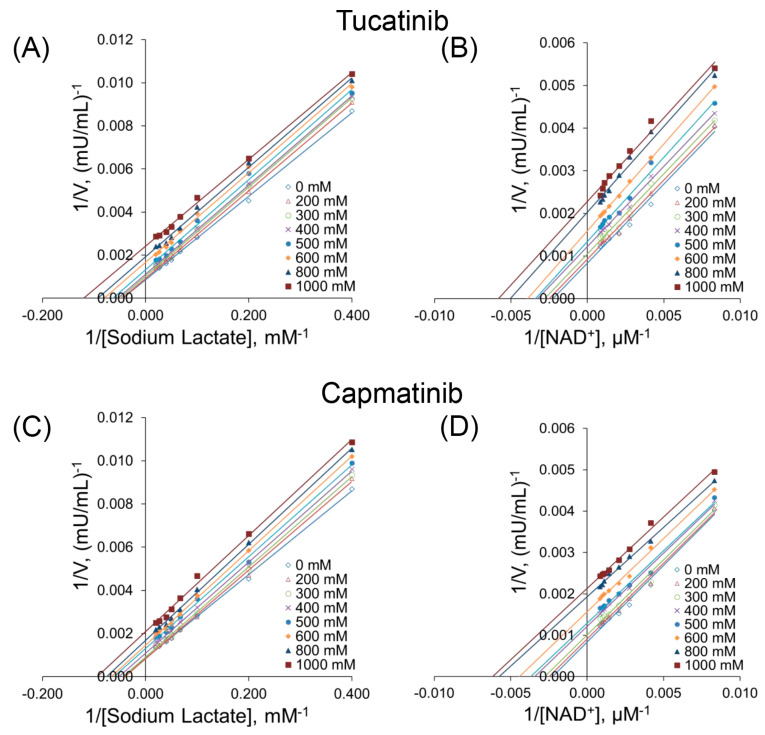
Kinetic studies of LDH-B in the presence of tucatinib (**A**,**B**) and capmatinib (**C**,**D**). LDH-B inhibition by the two compounds was assessed using various concentrations of sodium lactate (**A**,**C**) and NAD^+^ (**B**,**D**). Panels (**A**–**D**) show the Lineweaver plots of the kinetic data in Table 3 with nonlinear regression analysis.

We performed substrate competition assays to elucidate the mechanism by which both compounds inhibit the activity of LDH-B. We evaluated the LDH-B inhibitory activity of tucatinib and capmatinib at various concentrations of lactate and NAD^+^. The classic Μichaelis–Menten analysis also revealed that both compounds might inhibit LDH-B via the uncompetitive mechanism of inhibition. In detail, the K_m_ and V_max_ values for LDH-B were determined in the presence of increasing concentrations of the two candidate inhibitors (Table 2). As the concentration of both compounds increased, the V_max_ and K_m_ decreased (Table 2). At the same time, the Lineweaver plot lines were almost parallel (Figure 4), indicating uncompetitive inhibition, i.e., the inhibitors bind to the enzyme–substrate complex.

We then investigated if there was a time-dependent effect on the onset of inhibition by adjusting the duration for which tucatinib or capmatinib, and LDH-B were pre-incubated before commencing the enzymatic reaction. Notably, when LDH-B was pre-incubated with both compounds for 120 min, the inhibitory effect did not increase with longer pre-incubation times (Supplementary Figure S2).

We subsequently employed the mix-model inhibition fit to identify the mechanism of inhibition of LDH-B by the two compounds. Our analysis revealed that both compounds are preferably bound to the enzyme–substrate complex (i.e., the uncompetitive mechanism is most likely) without competing for binding to the active site with either lactate or NAD^+^, as indicated by the calculated α values (Table 3). Nevertheless, further experiments are needed to elucidate the mechanism by which these two compounds inhibit LDH-B.

### 3.4. Molecular Dynamics

**Figure 5:**
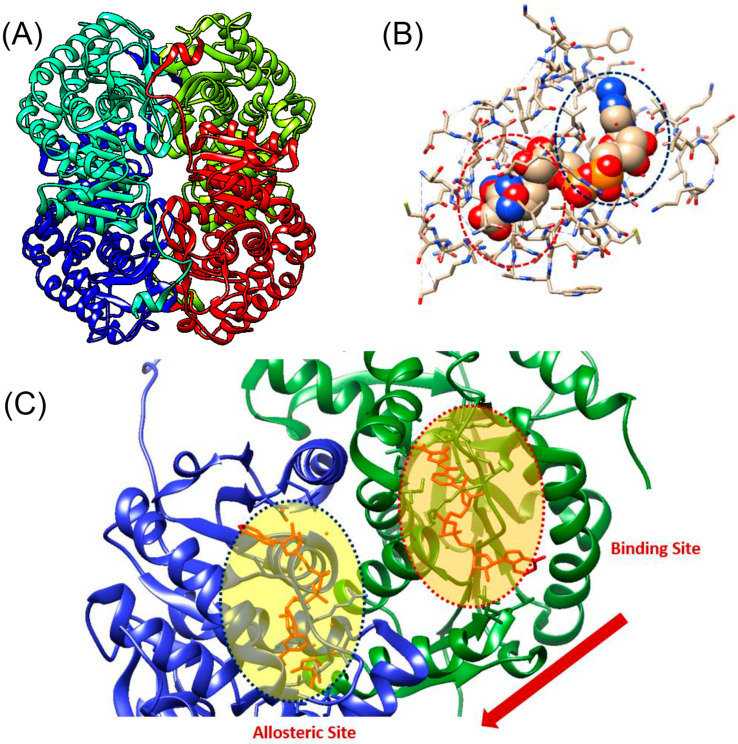
Docking and molecular dynamics studies of LDH-B with tucatinib and capmatinib. (**A**) The 3D structure of the LDH target used for docking studies was obtained from the protein data bank (1T2F:PDB). (**B**) Screenshots showing the interactions between LDH-B amino acid residues and either tucatinib (highlighted in red dot cycles) or capmatinib. (**C**) Representation of LDH-B in a complex with NAD^+^, which binds to the active site, and tucatinib (inhibitor), which binds to the allosteric site. NAD^+^ and tucatinib are shown as stick models in orange. The active site (binding site) and allosteric site are indicated in red-dot and blue-dot circles, respectively. Structural rearrangements by substrate binding are indicated by the red arrow.

We performed molecular dynamics studies to further evaluate the inhibitory effects of tucatinib and capmatinib and gather more information about their mechanisms of action (Figure 5). The 3D structure of the LDH-B tetramer is illustrated in Figure 5A. The catalytic site of LDH-B consists of His^193,^ which acts as a proton acceptor during the catalytic reaction; Arg^106,^ which stabilizes the substrate in the active site; Arg^169,^ which forms a salt bridge with Arg^106^ and helps to stabilize the enzyme–substrate complex; and Lys^102,^ which facilitates the binding of the coenzyme NAD^+^. Our analysis revealed that both compounds bind to the allosteric site, which is distant from the catalytic site (Figure 5B,C).

**Figure 6:**
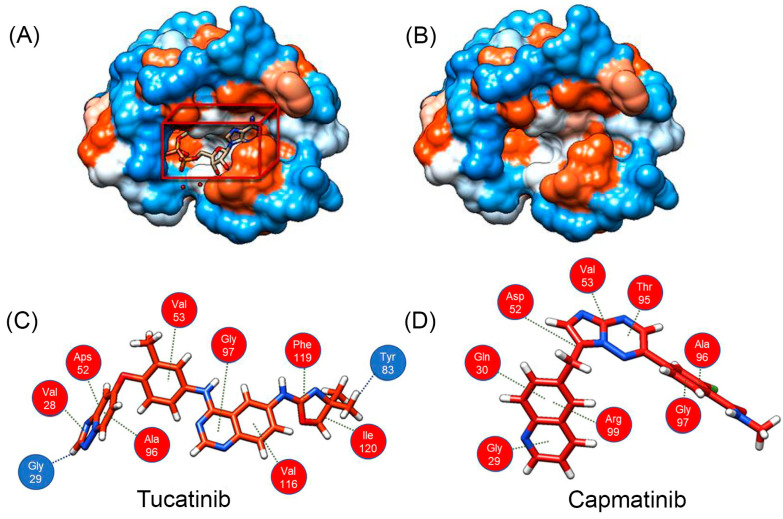
LDH-B contains an allosteric binding site, which is probably formed after the binding of lactate and NAD^+^. The allosteric binding site of LDH-B with (**A**) and without (**B**) tucatinib is shown. Tucatinib (**C**) interacts with hydrogen bonds and hydrophobic interactions with the amino acids of the active site, while capmatinib (**D**) only via hydrophobic interactions. The amino acids forming hydrogen bonds with tucatinib are highlighted in blue, while the amino acids interacting with both inhibitors through hydrophobic interactions are highlighted in red.

Furthermore, the allosteric site of LDH-B with and without tucatinib is illustrated in Figure 6A,B, respectively. More specifically, docking studies revealed that tucatinib interacts with the enzyme, forming two hydrogen bonds with the amino acids Gly^29^ and Tyr^83^ (Figure 6C). Additionally, tucatinib interacts with hydrophobic interactions (van der Waals forces) with the amino acids Val^28^, Asp^52^, Val^53^, Ala^96^, Gly^97^, Val^116^, Phe^119^, and Ile^120^. On the other hand, capmatinib interacts only with hydrophobic interactions with the amino acids Gly^29^, Gln^30^, Asp^52^, Val^53^, Thr^95,^ Ala^96^, Gly^97^, and Arg^99^ of the cavity (Figure 6D). Notably, our analysis and that of others [36] revealed that AXKO-0046 (binding energy −91.6 KJ/mol) interacts via van der Waals interactions with the amino acids Gly^29^, Gln^30^, Gly^97^, Val^98^, Arg99, Gln^101,^ and Ser^137^. The above data suggest that the LDH-B inhibitors bind to a unique allosteric binding site (or sites) on the enzyme, which is probably formed after the binding of the substrate and cofactor [36].

## 4. Discussion

Most cancer cells alter their metabolic pathways and prefer glycolysis over oxidative phosphorylation, a phenomenon known as the Warburg effect. LDH-A is a crucial enzyme in this phenomenon and a potential target for developing anticancer drugs [51]. On the other hand, the LDH-B isoform has been implicated in only specific types of cancer, including breast, colon, and lung [22,26], while it has been associated with aggressive cancer phenotypes [22,26]. To ensure the survival of glycolytic cancer cells, LDH-B is essential. Therefore, targeting lactate in oxidative cancer cells could provide a different chance to trigger necrosis in faraway glycolytic cancer cells that resist traditional anti-tumor therapies [52]. Studies have demonstrated that a complete hereditary deficiency of LDH-B has no consequences in humans [53,54]. Thus, inhibition of LDH-B may be an alternative strategy to fight cancer without causing other side effects. Despite the increasing recognition of the crucial role of LDH-B in the development of various cancer genotypes [5,10,22,26,29,55] there is still a limited amount of research focused on identifying potential inhibitors of this enzyme. Notably, although LDH-B is predominantly expressed in the heart muscle, its inhibition is unlikely to result in cardiovascular side effects. According to the information available at the National Library of Medicine (https://ghr.nlm.nih.gov/condition/lactate-dehydrogenase-deficiency (accessed on 1 February 2023)), a deficiency in LDH-B does not cause any health problems.

In this work, we demonstrated that two known drugs used for treating breast cancer (tucatinib) or lung cancer (capmatinib) might have a dual function and simultaneously inhibit enzymes necessary for the survival and metastasis of these types of cancer, including LDH-B and tyrosine kinase receptors. More specifically, tucatinib is an inhibitor of HER2, a part of the epidermal growth factor receptor family that is overexpressed in breast cancer [56]. Capmatinib is an inhibitor of the proto-oncogene c-Met (HGFR) that belongs to the tyrosine kinase family and has a significant role in the progression of lung cancers [57]. We subsequently employed the mixed-model fit to elucidate the mechanism by which tucatinib and capmatinib inhibit LDH-B activity (Table 3). Our analysis showed that both compounds exhibited an α value lower than 1, suggesting an uncompetitive mechanism of inhibition, i.e., the compounds preferentially bind to the enzyme–substrate complex. However, our results showed that prolonged pre-incubation of LDH-B with either of the two compounds did not affect their inhibitory effect. Furthermore, the classic Michaelis–Menten kinetic analysis verified that both compounds inhibit LDH-B through an uncompetitive mechanism of inhibition. This was determined by measuring the K_m_ and V_max_ values of LDH-B with increasing concentrations of the inhibitors. As the concentration of inhibitors increased, both K_m_ and V_max_ decreased. The Lineweaver plot lines were almost parallel, which suggests that the inhibitors bind to the enzyme–substrate complex (Figure 4). Our molecular dynamics studies supported the uncompetitive mechanism of inhibition for tucatinib and capmatinib, as both compounds interact with amino acids away from the enzyme’s active site via Van der Waals interactions (Figure 5).

Our work is vital for the following reasons: (i) To the best of our knowledge, the only LDH-B-specific inhibitor reported until the time of the publication of this work is AXKO-004 [36]. Most published studies that identified LDH inhibitors used the forward reaction (i.e., the conversion of pyruvate to lactate). However, most of these inhibitors are not specific to LDH-B; the authors of these studies primarily sought to confirm that the inhibitors are highly selective for LDH-A and used LDH-B for comparative purposes. It should be noted that there is a high structural similarity between LDH-B and LDH-A and a high structural homology of their catalytic sites (89% based on the NCBI basic local alignment search tool [BLAST]) [58]. However, LDH-B is an NAD^+^-dependent dehydrogenase that converts lactate to pyruvate, while it exhibits a higher affinity for lactate than pyruvate [59]. The differences in substrate preferences and the catalytic activity between the A and B LDH subunits (A subunit converts pyruvate to lactate while B facilitates the reverse reaction) are probably associated with a substitution of Ala by Gln residues (in the A and B subunits, respectively) located close to the binding site of the coenzyme phosphates [59]. However, studies aiming to identify selective inhibitors of LDH-B have used pyruvate as the substrate and NADH as a cofactor. For example, Shibita et al. [36] have recently identified the first specific uncompetitive inhibitor of human LDH-B, namely AXKO-0046, by using a RapidFire-Mass (RF-MS) system to monitor the conversion of NADH to NAD^+^. Another known LDH-B inhibitor is oxamate, a pyruvate analog that competes with the substrate (pyruvate) for binding to the active site [60]. In our work, we used lactate and NAD^+^ as substrates and cofactors, respectively, to identify inhibitors that selectively inhibit the conversion of lactate to pyruvate. The mechanism of LDH-B inhibition by tucatinib and capmatinib was examined using increasing concentrations of lactate and NAD^+^. Both compounds were evaluated at eight different lactate and NAD^+^ concentrations. The mix-model inhibition fit analysis revealed that both compounds are preferably bound to the enzyme–substrate complex, suggesting an uncompetitive inhibitory mechanism concerning lactate and NAD^+^ (Table 3)_._ The uncompetitive mechanism of action of tucatinib and capmatinib on LDH-B was further supported by the parallel Lineweaver–Burk plots obtained following the classic Michaelis–Menten analysis when LDH-B was assayed in the presence of increasing concentrations of both compounds (Figure 4). It should be pointed out that we have not performed any cytotoxic assays, as both compounds are commercially available drugs. Although a relatively high concentration (i.e., at mM levels) of both compounds is required to inhibit LDH-B, our findings can serve as a starting point for identifying more potent inhibitors of this enzyme. Notably, the concentrations greater than 0.5 mM for tucatinib and capmatinib used in our assays are within the safe range (https://www.drugs.com/ (accessed on 31 March 2023)). In detail, the dose of tucatinib, combined with trastuzumab and capecitabine, is 300 mg (0.625 mM) for treating breast cancer. In addition, for the treatment of non-small lung cancer, the adult dose of capmatinib is 400 mg (0.97 mM). Therefore, these compounds could be promising candidates for further evaluation as LDH-B inhibitors in cancer therapy.

(ii) Furthermore, to our knowledge, a high-throughput screening (HTS) assay for the identification of LDH-B-specific inhibitors using the reverse reaction (lactate to pyruvate) has not yet been reported. The activity of several NAD^+^-dependent dehydrogenases, including LDH-B, is usually measured by monitoring the formation of NADH at 340 nm; this approach is unsuitable for HTS assays aiming to identify hit and lead compounds from chemical libraries as many compounds absorb at the UV range. Other methods measure the rate of NADH formation and disappearance by measuring the fluorescence of NADH, which exhibits characteristic excitation and emission maxima at 340 nm and 480 nm, respectively [61,62]. Nevertheless, it is essential to note that this method can produce erroneous results, both false positives and false negatives, due to the potential for fluorescence interference at the excitation and emission wavelengths of NADH.

(iii) The conversion of pyruvate to lactate by LDH-A can also be monitored using a label-free assay of RF-MS [63]. However, this method was exclusively employed for secondary assays, which were conducted to confirm the chosen compounds after the initial screening procedure utilizing the fluorescence assay. Recently, Shibita et al. [36] reported an RF-MS assay to monitor the conversion of NADH to NAD^+^ using LDH activities in an HTS format. Although the assay showed excellent performance, with an average Z’ factor > 0.65, it requires specific equipment. We have recently optimized a high-throughput NBT/PMS-based colorimetric assay using the Design of Experiments to determine LDH-B activity in 96-well format at the early stages of drug discovery with a Z′ factor of 0.84 [46]. However, we have not tested whether the assay is suitable for identifying potential inhibitors of LDH-B. Herein, we also validated our assay using AXKO-0046, a selective inhibitor of LDH-B, and demonstrated that this approach is ideal for identifying potential inhibitors of LDH-B and other NAD^+^-dependent hydrogenases. Our approach is fast and accurate and requires only a multi-plate reader. In conclusion, the LDH-B assay we developed to monitor LDH-B activity has several advantages over current assay methods.

## 5. Conclusions

LDH-B is an essential target for the development of inhibitors for cancer treatment. To the best of our knowledge, this is the first instance where the reverse reaction orchestrated by LDH, i.e., the conversion of lactate to pyruvate, has been employed to identify compounds that selectively inhibit LDH-B. At the time of this work’s publication, only one specific LDH-B inhibitor, AXKO-0046, had been reported. Interestingly, this compound was identified using the forward reaction, which involves the conversion of pyruvate to lactate. In this study, we spotlighted two commercially available drugs, tucatinib and capmatinib, showcasing their potential as dual inhibitors of enzymes critical to the progression of certain cancers. Our in vitro assessments elucidate that both compounds inhibit LDH-B activity through an uncompetitive mechanism, a finding further substantiated by our docking studies. A significant contribution of our endeavor is the validation of an HTS colorimetric assay, previously developed in our laboratory, adept at recognizing potent LDH-B antagonists. This research paves the way for developing LDH-B inhibitors as promising interventions against specific malignancies, notably breast and lung cancers. To build on these preliminary insights, subsequent investigations employing cell-based assays with breast and lung cell cultures are imperative. These will illuminate the precise mechanistic dynamics of the flagged inhibitors and could steer efforts toward lead optimization and pre-clinical assessments. Exploring derivatives and gauging their safety and efficacy could potentially augment the current roster of LDH-B inhibitors.

## Figures and Tables

**Table 1:** Properties of potential LDH-B inhibitors and AXKO-0046 (positive control).

#	Compound	CLogP ^1^	Molecular Mass (g/mole)	Binding Affinity ^2^
1	Tucatinib	5.32	480.52	−9.2
2	Capmatinib	1.88	412.42	−9.3
3	Moxidectin	6.65	639.80	−9.5
4	Rifampicin	3.86	697.77	−9.5
5	Acetyldigoxin	3.70	806.98	−10.0
6	Bictegravir	−0.01	449.38	−9.0
7	Calicheamicin	2.00	1368.65	−9.3
8	Capreomycin	N.A. ^3^	1321.41	−9.1
9	AΧKO-0046	4.74	448.47	−6.6

^1^ Calculated with Chem Draw v12.0; ^2^ Calculated with Auto Dock Vina; ^3^ N.A.: not available.

**Table 2:** Effect of Tucatinib and Capmatinib on K_m_ and V_max_ values of lactate and NAD^+^ in LDH-B activity *.

	Tucatinib, (μΜ)							
	Control	200	300	400	500	600	800	1000
**Lactate**								
V_max_ (mU/mL)	1052 ± 36	971 ± 26	916 ± 22	858 ± 17	772 ± 26	644 ± 19	527 ± 16	421 ± 23
K_m_ (mΜ)	18.5 ± 1.6	17.6 ± 0.9	17.2 ± 1.2	16.5 ± 1.1	15.9 ± 1.3	14.3 ± 0.9	10.9 ± 0.7	8.7 ± 0.8
**NAD^+^**								
V_max_ (mU/mL)	1035 ± 42	983 ± 41	893 ± 31	784 ± 41	723 ± 32	624 ± 26	521 ± 21	474 ± 25
K_m_ (μΜ)	311 ± 21	305 ± 12	271 ± 21	254 ± 12	220 ± 17	209 ± 14	178 ± 16	168 ± 18
	**Capmatinib, (μM)**							
	Control	200	300	400	500	600	800	1000
**Lactate**								
V_max_ (mU/mL)	1052 ± 36	987 ± 42	927 ± 33	839 ± 28	725 ± 26	653 ± 21	587 ± 16	513 ± 19
K_m_ (mΜ)	18.5 ± 1.6	17.1 ± 1.4	16.5 ± 1.2	15.8 ± 1.1	14.5 ± 0.9	13.3 ± 0.8	12.6 ± 0.9	12.4 ± 1.2
**NAD^+^**								
V_max_ (mU/mL)	1035 ± 42	973 ± 23	892 ± 26	799 ± 22	733 ± 31	629 ± 26	526 ± 27	469 ± 32
K_m_ (μΜ)	311 ± 21	308 ± 23	298 ± 18	286 ± 21	279 ± 15	256 ± 16	245 ± 14	211 ± 18

* Data are presented as mean values ± SD (n = 3).

**Table 3:** LDH-B inhibition data obtained with tucatinib and capmatinib with lactate and NAD^+^.

Parameter	Tucatinib		Capmatinib	
	NAD^+^	Lactate	NAD^+^	Lactate
Κi (mM)	4.16	2.96	1.53	2.69
α ^a^	0.20	0.28	0.58	0.36
r^2 b^	0.9648	0.9743	0.9630	0.9815

^a^ The value of the alpha (α) factor is indicative of the mechanism of inhibition as described in the text; ^b^ Goodness of fit.

## Data Availability

The data generated in this study are available upon request by contacting the corresponding author.

## Supplementary Materials

The following supporting information can be downloaded at: https://www.mdpi.com/article/10.3390/pharmaceutics15102411/s1. Figure S1: Effect of potential LDH-B inhibitors on the formation of blue-purple formazan; Figure S2: Effect of pre-incubation time on the inhibitory activity of tucatinib (A) and capmatinib (B) on LDH-B activity; Table S1: RMSD values of the eight best candidates and free energies of their complexes with the enzyme.
