# Supplementary material for: Targeting Lactate Dehydrogenase-B as a Strategy to Fight Cancer: Identification of Potential Inhibitors by In Silico Analysis and In Vitro Screening
Source: Pharmaceutics. 2023 Oct 1;15(10):2411. doi: 10.3390/pharmaceutics15102411 (PMC10609963; doi:10.3390/pharmaceutics15102411)
Supplement: Supplementary file 1 [file pharmaceutics-15-02411-s001.zip › pharmaceutics-2602551-supplementary.pdf]

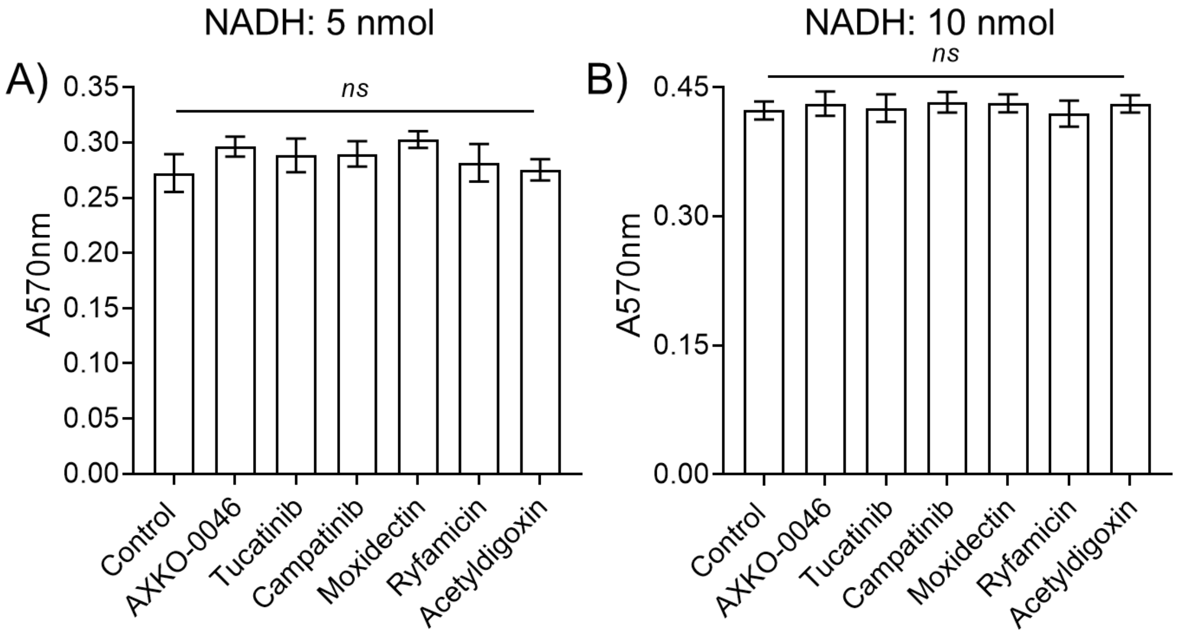

Supplementary Figure S1. Effect of potential LDH-B inhibitors on the formation of blue-purple formazan. 1000 µM of each compound were mixed with either 5 nmol (A) or 10 nmol (B) of NADH in a final volume of 10 µL in the wells of a 96-well plate. Subsequently, 90 µL of 50 mM CHES buffer, pH 9.6, supplemented with 150 mM NaCl, 300 µM nitroblue tetrazolium (NBT), 30 µM phenazine methosulfate (PMS), and 0.13% gelatin. Control wells contained none of the inhibitors. The mixtures were incubated for 5 min at 25 ^{o}C, and the absorbance at 570 nm was measured. As shown, the presence of all five tested compounds did not interfere with forming the blue-purple formazan derivative. The results are presented as mean values ± S.D. (n=3). One-way ANOVA, followed by Dunnett's test for multiple comparisons, was used for statistical analysis. $p<0.05$ was considered to be statistically significant. n.s.: not-significant.

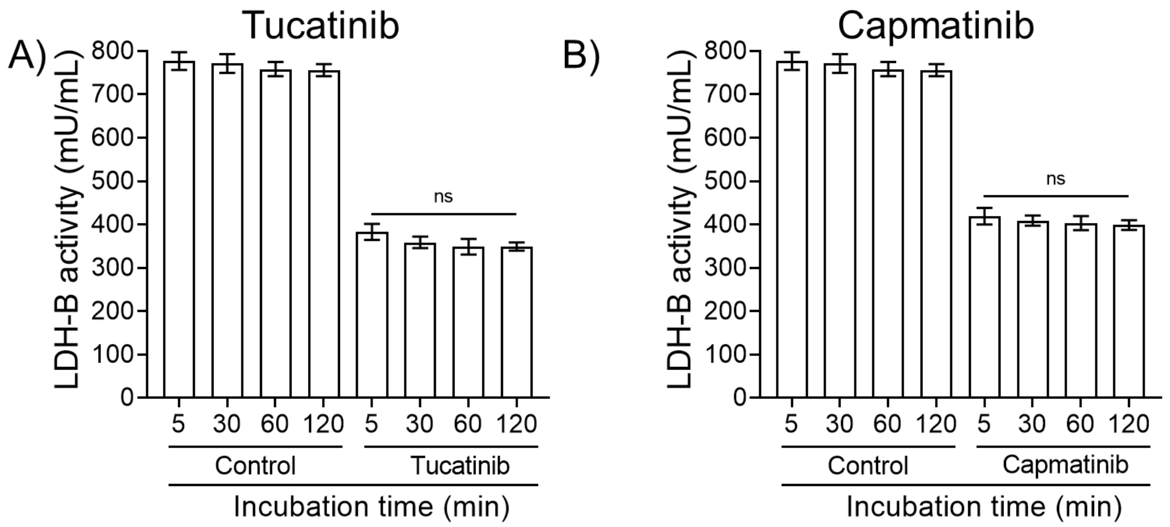

Supplementary Figure S2. Effect of pre-incubation time on the inhibitory activity of tucatinib (A) and capmatinib (B) on LDH-B activity. Each of the two compounds (1000 µM) was incubated with 2.5 nmol of LHD-B in a final volume of 10 µL in the wells of a 96-well plate at the indicated times. Subsequently, 80 µL of 50 mM CHES buffer, pH 9.6, supplemented with 150 mM NaCl, 300 µM nitroblue tetrazolium (NBT), 30 µM (phenazine methosulfate) PMS, and 0.13% gelatin. The reaction was initiated by adding 10 µL of substrate mix (10 mM NAD+ and 250 mM sodium lactate), and LDH-B activity was determined as indicated in the text. The control wells contained none of the inhibitors. The results are presented as mean values ± S.D. (n=3). One-way ANOVA, followed by Dunnett's test for multiple comparisons, was used for statistical analysis. p<0.05 was considered to be statistically significant. n.s.: not-significant.

Supplementary Table S1. RMSD* values of the eight best candidates and free energies of their complexes with the enzyme.

#	Compound	RMSD (Å)	Free Energy ΔG (kcal/mol)
1	Tucatinib	0.82	-14.42
2	Capmatinib	0.80	-16.21
3	Moxidectin	1.15	-6.34
4	Rifampicin	0.99	-14.01
5	Acetyldigoxin	1.21	-5.88
6	Bictegravir	1.14	-6.01
7	Calicheamicin	1.55	-4.87
8	Capreomycin	0.89	-15.12

*RMSD: Root mean square deviation
